# Do perceptions of harm and addictiveness influence adolescent's willingness to use various tobacco and nicotine products?

**DOI:** 10.18332/tpc/204746

**Published:** 2025-06-27

**Authors:** Melissa A. Little, Indika Mallawaarachchi, Asal Pilehvari, Ponni Velmurugan, Abigail G. Wester, Kara P. Wiseman

**Affiliations:** 1 Department of Public Health Sciences, School of Medicine, University of Virginia, Charlottesville, United States; 2 Comprehensive Cancer Center, University of Virginia, Charlottesville, United States; 3 Division of Community Health Sciences, School of Public Health, University of Illinois Chicago, Chicago, United States

**Keywords:** prevention, adolescents, susceptibility, cessation, willingness, tobacco and nicotine products

## Abstract

**INTRODUCTION:**

The Theory of Reasoned Action has been widely used to explain adolescent tobacco and nicotine product (TNP) use, focusing on intentions and subjective norms. However, the ‘reactive pathway’, emphasizing situational influences and willingness to use, better predicts TNP use in adolescents. While prior research has examined willingness for cigarettes and e-cigarettes, its application to the broader range of available TNPs is limited. This study investigates adolescent characteristics across varying levels of TNP use willingness. We hypothesized that perceived harm and addictiveness would be associated with willingness to use tobacco.

**METHODS:**

Secondary school students aged 14–15 years (n=348) completed a survey that assessed demographics and TNP use history, willingness to use TNPs, peer use, and perceived harm and addictiveness of TNPs. Descriptive statistics were used to characterize the population overall and by willingness to use TNPs. Multivariable logistic regression models estimated associations between TNP-specific willingness to use, gender, race, ethnicity, and peer use with TNP-specific perceived harm and addictiveness.

**RESULTS:**

Across the TNPs, 22.1% were current users, 23.3% were willing non-users and 54.7% were non-willing non-users. Significant differences in perceived harm by willingness were for cigarettes, e-cigarettes, and hookah, while perceptions of addictiveness varied by willingness group for all TNPs with the exception of cigarillos (all p<0.05). Willing non-users had lower odds of perceived addictiveness (smokeless tobacco, OR=0.29; 95% CI: 0.11–0.81; cigar, OR=0.33; 95% CI: 0.15–0.70) and harm (e-cigarettes, OR=0.38; 95% CI: 0.19–0.76; pipe, OR=0.41; 95% CI: 0.17–0.98; cigarillos/little cigars, OR=0.34; 95% CI: 0.12–0.92; cigars, OR=0.24; 95% CI: 0.11–0.54) compared to non-willing non-users.

**CONCLUSIONS:**

Adolescents have varying levels of susceptibility to using TNPs. In order to develop effective interventions for adolescents, the diverse range of available TNPs with specific risks and appeal need to be considered.

**CLINICAL TRIAL REGISTRATION:** The study is registered on the official website of ClinicalTrials.gov

**IDENTIFIER:** ID NCT05396911

## INTRODUCTION

In 2023, 12.6% of high school students (aged 14–18 years) and 6.6% of middle school students (aged 11–14 years) reported current tobacco and nicotine product (TNP) use in the United States (US)^1^. E-cigarettes remain the most commonly used product, however 30.5% of high school tobacco users report using a combustible product, and 31.0% report using multiple products^1^. Multiple TNP use is associated with higher nicotine dependence and greater difficulty quitting in youth^2^. Any nicotine exposure places youth at increased risk for learning, memory and attention deficits, mood disorders, impulsivity and other drug addiction^3^, and e-cigarettes have negative cardiovascular effects including elevated heart rate and diastolic blood pressure^4^.

While research indicates that the majority of young people who use TNPs want to quit within a short time of starting usage^5^, nicotine addiction occurs very quickly, making the odds of a successful quit attempt low^6^. Adolescents who begin smoking before the age of 21 years are also less likely to quit compared to those starting after this age, highlighting the need for effective TNP prevention and cessation programs for youth^7^. However, before such interventions can be developed, there is a need for a greater understanding of the drivers of TNP use and non-use, particularly given the rise of new products among adolescents often promoted using safer health appeals, such as e-cigarettes^8,9^.

The Theory of Reasoned Action^10^ has been used extensively to explain TNP behaviors among youth^11-14^. The Theory of Reasoned Action suggests that one’s intention to engage in a behavior is influenced by their perceived behavioral control, attitudes, and subjective norms, which ultimately influences their behaviors^15^. Researchers have found that intentions to use TNP predict future use, while subjective norms (such as perceived peer use) predict TNP cessation among adolescents who report a history of TNP use^16,17^. However, this approach has been criticized because adolescent decision making is often influenced by social influences and reactions to situations, referred to as the ‘reactive pathway’^18^. The Prototype/Willingness model suggests that adolescent risk behavior is based on a dual-processing approach: a social reaction path, favoring behavioral willingness, and a reasoned path in which attitudes and subjective norms predict intentions to engage in a behavior, similar to the Theory of Reasoned Action^10,19^. The social reaction pathway is often measured as ones willingness to engage in a behavior when the substance is available^20^. Previous studies have shown that willingness is a better predictor of adolescent cigarette and e-cigarette use compared to intentions^18,21-23^. However, we are not aware of any studies that have examined willingness to use the range of currently available TNPs among adolescents.

As such, little is also known regarding how adolescents’ willingness to use TNPs is associated with their perceptions of harm and addictiveness across products. Perceptions of harm associated with TNP use are higher among non-using youth compared to youth that report TNP use behaviors^24,25^. However, less research has been done examining tobacco risk perceptions, such as harm and addictiveness, across the full range of available TNPs. Given that youth who are more willing to use TNPs likely underestimate the harm and addictiveness of TNPs, it is critical to examine how the perceptions of these groups of youth vary by product to inform future intervention efforts.

The purpose of this study was to understand characteristics of TNP willingness group affiliation among adolescents. Additionally, we sought to gain a deeper understanding of how attitudes, beliefs, and subjective norms of TNPs varied by willingness group affiliation among adolescents, to inform the development of programs that address the wide range of currently available TNPs, and contemporary patterns of use.

## METHODS

### Participants and procedures

Participants were 405 US 9th grade students, adolescents aged 14–15 years, from 11 public high schools in the South and South-Central regions of Virginia who participated in an anonymous assessment in the form of a paper and pencil survey in Spring 2022. Two weeks prior to administering the assessment, students were provided with an opt-out form to bring home and discuss with their parents. Any students who did not want to participate were instructed to have their parents sign the opt-out form and return it to their teacher. On the date of the assessment, any student who opted out of the study was sent to another classroom for the duration of the class period (n=3). Additionally, some students were unavailable (n=20) or absent (n=39), and therefore not included. The final sample included 348 students (84.9% response rate). Student participation in classroom procedures indicated student assent to participate. Data collection took place in the classroom over one 90-minute class period and was administered by trained program staff who explained the survey procedures. This study was approved by the Social Behavioral Sciences Institutional Review Board at the University of Virginia. All students received a $5 gift card as a thank you for their participation.

### Measures

The survey assessed demographics (sex, race, and ethnicity), TNP use history, willingness to use TNPs, peer use of TNPs and perceived harm and addictiveness of TNPs. Each question asking about TNP use behaviors and/or TNP-related beliefs included responses for each of the following TNPs: cigarettes/roll-your-own, smokeless tobacco/snus, e-cigarettes, pipe filled with tobacco, cigarillos/little cigars, cigars and hookah. Current use of TNPs was assessed by asking students: ‘In the past 30 days, did you use the following tobacco products…?’. Response options included: every day, some days, and not at all. Current TNP use was defined as a student using a TNP every day or some days in the past 30 days. Among students who did not use TNPs, willingness to use TNPs was measured through three items for each TNP. The questions asked: ‘If offered the following tobacco products, how willing would you be to: 1) Take one puff/pinch or pouch; 2) Smoke a whole [tobacco product]/use a pinch or pouch; and 3) take one to try later?’ Responses were on 4-point scales with response points: not at all willing (1) to very willing (4). A composite willingness score was created for each product by summing the three items^23^. To be classified as non-willing to use a TNP, students had to report ‘not at all willing’ to each of the willingness questions (total score of 3 per product). All other responses were coded as willing to use.

Peer use was assessed by asking: ‘Of your five closest friends, how many of them use any kind of tobacco?’ with response options ranging from 0 to 5. Perceived harm was assessed by asking students: ‘Overall, how harmful (bad for your body) do you think each of the following is?’ with response options on a 3-point scale with anchor points ‘not at all harmful’ to ‘very harmful’ and ‘I don’t know’. Responses were also dichotomized to ‘very harmful’ or other responses. Perceived addictiveness was assessed by asking students: ‘Overall, how addictive (hard to quit) do you think each of the following is?’ for each tobacco product with response options on a 3-point scale with anchor points ‘not at all addictive’ to ‘very addictive’ and ‘I don’t know’. Responses were dichotomized to ‘very addictive’ or other responses.

### Data analysis

Descriptive statistics were used to characterize the population overall and by willingness to use TNPs. Differences in demographics, TNP use characteristics, perceived harm, and perceived addictiveness were compared by willingness to use any TNP using chi-squared tests. Significance was set at p<0.05; for tests comparing perceived harm and perceived addictiveness across products and by willingness to use TNPs, significance was adjusted for multiple comparisons using Bonferroni correction. Multivariable logistic regression models estimated associations between TNP-specific willingness to use, sex, race, ethnicity, and peer use with TNP-specific perceived harm and addictiveness. Models provided odds ratios (ORs) and 95% confidence intervals.

## RESULTS

The sample of participants aged 14–15 years was mostly White (65.5%), non-Hispanic (87.8%), and 53.5% were male (Table 1). Additionally, 35.0% reported using at least one TNP in their lifetime and 22.1% reported past 30-day use of at least one TNP, while, 54.7% were classified as non-willing non-users, and 23.3% as willing non-users. E-cigarettes were the most commonly reported product used in the past 30 days (93.4%), followed by cigarettes (30.3%), smokeless tobacco (22.4%), cigars (19.7%), cigarillos/little cigars (19.7%), and pipe (11.8%).

**Table 1:** Sample characteristics by willingness to use tobacco and nicotine products among secondary school students aged 14–15 years in the United States (N=344)

*Characteristics*	*Total* *n (%)*	*Non-willing non-user* *n (%)*	*Willing non-user* *n (%)*	*Current user* *n (%)*
**Total**	344 (100)	188 (54.7)	80 (23.3)	76 (22.1)
**Gender**				
Male	178 (53.5)	109 (59.2)	41 (51.9)	28 (40.0)
Female	155 (46.5)	75 (40.8)	38 (48.1)	42 (60.0)
**Hispanic/Latino**	41 (12.2)	20 (10.9)	11 (13.9)	10 (13.9)
**Race**				
Black	56 (17.0)	27 (14.8)	10 (12.8)	19 (27.1)
White	216 (65.5)	124 (68.1)	55 (70.5)	37 (52.9)
Other	27 (8.2)	15 (8.2)	4 (5.1)	8 (11.4)
Multiple	31 (9.4)	16 (8.8)	9 (11.5)	6 (8.6)
**Lifetime tobacco use^a^**	120 (35.0)	16 (8.5)	28 (35.4)	76 (100.0)
**Tobacco and nicotine products^b^**				
Cigarettes	53 (44.2)	7 (43.8)	8 (28.6)	38 (50.0)
Smokeless tobacco	31 (25.8)	0 (0.0)	5 (17.9)	26 (34.2)
E-cigarettes	109 (90.8)	11 (68.8)	23 (82.1)	75 (98.7)
Pipe	18 (15.0)	0 (0.0)	2 (7.1)	16 (21.1)
Cigarillos/little cigars	29 (24.2)	2 (12.5)	1 (3.6)	26 (34.2)
Cigars	32 (26.7)	2 (12.5)	4 (14.3)	26 (34.2)
**Peer use** (number of friends)				
0	164 (49.4)	126 (70.0)	31 (38.8)	7 (9.7)
1	47 (14.2)	16 (8.9)	19 (23.8)	12 (16.7)
2	36 (10.8)	14 (7.8)	12 (15.0)	10 (13.9)
3–5	85 (25.6)	24 (13.3)	18 (22.5)	43 (59.7)

Pearson’s chi-squared test was used to compare the differences of characteristics among 3 user groups. All statistical tests are significant at p<0.05 with the exception of race and Hispanic/Latino ethnicity.

a Prevalence among user group.

b Prevalence among lifetime tobacco users.

**Figure 1:**
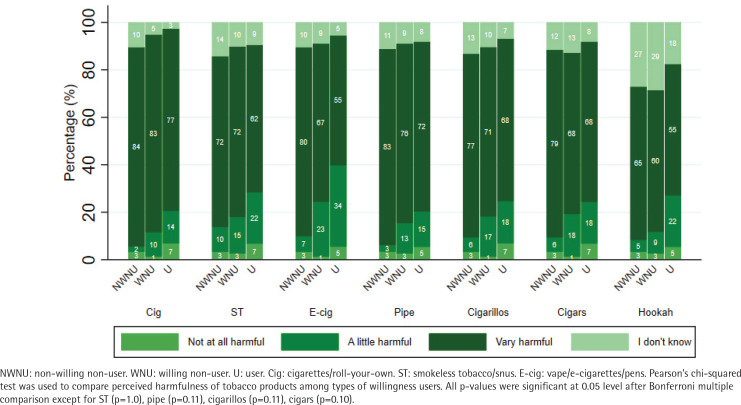
Perceived harmfulness of tobacco and nicotine products by willingness group affiliation among adolescents aged 14–15 years and enrolled in secondary school in the United States (N=344)

**Figure 2:**
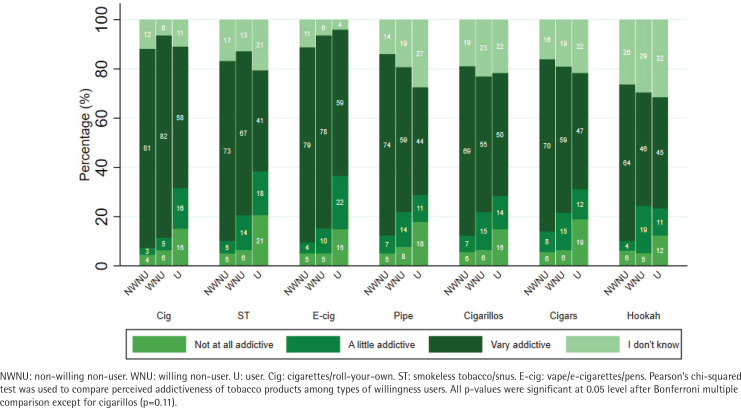
Perceived addictiveness of tobacco and nicotine products by willingness group affiliation among adolescents aged 14–15 years and enrolled in secondary school in the United States (N=344)

Perceived harm varied across TNPs and there were significant differences in perceived harm by willingness to use cigarettes, e-cigarettes, and hookah (Figure 1). Specifically, non-willing non-users had higher reported perceived harm for cigarettes, e-cigarettes, and hookah, followed by willing non-users. Current TNP users had significantly lower perceived harm for all three products, with the lowest perceived harm for e-cigarettes and hookah (55% reporting ‘very harmful’ for each product). Perceived addictiveness varied across products and there were significant differences in perceived addictiveness by willingness to use for all products except cigarillos (Figure 2). For non-willing non-users, perceived addictiveness was highest for cigarettes (81%), followed by e-cigarettes (79%), and pipe (74%). For willing non-users, perceived addictiveness was highest for cigarettes (82%), followed by e-cigarettes (78%), and smokeless tobacco (67%). For current TNP users, perceived addictiveness was highest for e-cigarettes (59%), followed by cigarettes (58%) and cigarillos (50%). Responses of ‘I don't know’ were more common for perceived addictiveness (range: 4.1–31.5%) than for perceived harm (range: 2.7–28.6%).

Associations between product specific willingness to use and perceived harm varied by product (Table 2). Compared to non-willing non-users, willing non-users for e-cigarettes, pipe, cigarillos, and cigars had lower odds of perceived harm for each product (all p<0.05). Current users had lower odds of perceived e-cigarette harm compared to non-users (OR=0.29; 95% CI: 0.13–0.63). Demographic correlates of perceived harm varied by product. For smokeless tobacco, pipe, cigarillos, and cigars, male students had lower odds of perceived harm of each product compared to female students (all p<0.05). Hispanic students had lower odds of perceived harm for every tobacco product except for e-cigarettes compared to non-Hispanic students (all p<0.05).

**Table 2:** Associations between perceived harm of tobacco and nicotine products among secondary school students aged 14–15 years in the United States (N=344)

	*Cigarettes* *OR (95% CI)*	*Smokeless* *tobacco* *OR (95% CI)*	*E-cigarettes* *OR (95% CI)*	*Pipe* *OR (95% CI)*	*Cigarillos/little* *cigars* *OR (95% CI)*	*Cigars* *OR (95% CI)*
**Willingness user group** (ref: non-willing non-user)						
Willing non-user	0.79 (0.34–1.82)	0.53 (0.19–1.50)	0.38 (0.19–0.76)*	0.41 (0.17–0.98)*	0.34 (0.12–0.92)*	0.24 (0.11–0.54)*
User	0.43 (0.13–1.37)	0.43 (0.14–1.30)	0.29 (0.13–0.63)*	0.54 (0.10–2.79)	1.09 (0.25–4.69)	0.43 (0.12–1.52)
**Male** (ref: female)	0.56 (0.29–1.10)	0.58 (0.34–0.97)*	0.67 (0.39–1.17)	0.43 (0.23–0.80)*	0.35 (0.19–0.65)*	0.44 (0.25–0.80)*
**Race** (ref: White)						
Black	0.66 (0.29–1.51)	0.65 (0.32–1.31)	0.58 (0.29–1.16)	0.53 (0.24–1.13)	0.40 (0.19–0.83)*	0.50 (0.24–1.02)
Other	1.44 (0.54–3.88)	1.20 (0.55–2.61)	1.88 (0.79–4.45)	1.48 (0.57–3.82)	1.91 (0.73–4.98)	3.41 (1.20–9.71)
**Hispanic/Latino** (ref: non-Hispanic/Latino)	0.25 (0.10–0.66)*	0.37 (0.16–0.88)*	0.41 (0.16–1.02)	0.25 (0.10–0.66)*	0.16 (0.06–0.42)*	0.17 (0.06–0.47)*
**Peer use** (number of friends) (ref: 0 friends)						
1–2	0.69 (0.31–1.54)	1.45 (0.74–2.83)	1.03 (0.50–2.09)	1.21 (0.55–2.67)	0.93 (0.45–1.93)	1.07 (0.51–2.22)
3–5	0.87 (0.37–2.04)	0.97 (0.50–1.87)	0.86 (0.41–1.81)	0.69 (0.33–1.43)	0.94 (0.45–1.99)	0.64 (0.31–1.30)

Each column provides results from one multivariable logistic regression model, which examined the association between willingness user groups and dichotomized perceived harm of each tobacco and nicotine products (very harmful vs rest), adjusting for sex, race, ethnicity, and peer tobacco use.

* Statistical significance at p<0.05.

Associations between product-specific willingness to use and perceived addictiveness varied by product (Table 3). Specifically, willing non-users for smokeless tobacco and cigars had lower odds of perceiving each product to be very addictive compared to non-willing non-users of each product (OR=0.29; 95% CI: 0.11–0.81 and OR=0.33; 95% CI: 0.15–0.70 for smokeless tobacco and cigars; respectively). Current users had lower odds of perceived addictiveness for e-cigarettes compared to non-willing non-users (OR=0.44; 95% CI: 0.20–0.96). Demographic correlates of perceived addictiveness also varied by product. Peer use was associated with perceived addictiveness for pipe and cigarillos only. Specifically, compared to students with no friends who use TNPs, those with TNP-using friends had lower odds of perceived addictiveness for pipe (OR=0.46; 95% CI: 0.25–0.84, and OR=0.46; 95% CI: 0.25–0.86 for 1–2 and 3–5 tobacco using friends; respectively). Students with 3–5 TNPs-using friends had lower odds of perceived addictiveness for cigarillos and cigars compared to students with no TNP-using friends (OR=0.47; 95% CI: 0.25–0.89 for cigarillos and OR=0.50; 95% CI: 0.26-0.94 for cigars).

**Table 3:** Associations between perceived addictiveness of tobacco and nicotine products among secondary school students aged 14–15 years in the United States (N=344)

	*Cigarettes* *OR (95% CI)*	*Smokeless* *tobacco* *OR (95% CI)*	*E-cigarettes* *OR (95% CI)*	*Pipe* *OR (95% CI)*	*Cigarillos/little* *cigars* *OR (95% CI)*	*Cigars* *OR (95% CI)*
**Willingness user group** (ref: non-willing non-user)						
Willing non-user	0.85 (0.41–1.78)	0.29 (0.11–0.81)*	0.87 (0.40–1.88)	0.47 (0.22–1.04)	0.60 (0.24–1.47)	0.33 (0.15–0.70)*
User	0.52 (0.18–1.51)	0.74 (0.23–2.35)	0.44 (0.20–0.96)*	1.34 (0.28–6.41)	1.33 (0.37–4.77)	1.12 (0.33–3.89)
**Male** (ref: female)	0.76 (0.43–1.37)	0.77 (0.46–1.30)	0.63 (0.36–1.12)	0.66 (0.40–1.11)	0.59 (0.35–0.97)*	0.51 (0.30–0.85)*
**Race** (ref: White)						
Black	0.30 (0.15–0.60)*	0.43 (0.22–0.85)*	0.38 (0.19–0.76)*	0.43 (0.22–0.84)*	0.32 (0.16–0.63)*	0.36 (0.18–0.70)*
Other	1.05 (0.44–2.50)	0.81 (0.39–1.68)	0.81 (0.37–1.80)	0.87 (0.41–1.84)	0.76 (0.37–1.56)	0.71 (0.34–1.48)
**Hispanic/Latino** (ref: non-Hispanic/Latino)	0.39 (0.16–0.98)*	0.41 (0.18–0.98)*	0.51 (0.21–1.24)	0.62 (0.26–1.46)	0.43 (0.18–1.01)	0.53 (0.22–1.30)
**Peer use** (number of friends) (ref: 0 friends)						
1–2	0.60 (0.29–1.23)	0.72 (0.38–1.36)	0.82 (0.39–1.71)	0.46 (0.25–0.84)*	0.58 (0.31–1.08)	0.56 (0.30–1.05)
3–5	0.60 (0.29–1.26)	0.59 (0.31–1.12)	0.64 (0.30–1.37)	0.46 (0.25–0.86)*	0.47 (0.25–0.89)*	0.50 (0.26–0.94)*

Each column provides results from one multivariable logistic regression model, which examined the association between willingness user groups and dichotomized perceived addictiveness of each tobacco and nicotine products (very addictive vs rest), adjusting for sex, race, ethnicity, and peer tobacco use.

* Statistical significance at p<0.05.

## DISCUSSION

The current study explored willingness, perceived harms and perceived addictiveness across seven TNPs in a group of adolescents aged 14–15 years. As hypothesized, willingness to use tobacco was associated with perceptions of harm and addictiveness, but patterns differed by TNP. Additionally, peer use, sex, race, and ethnicity were associated with perceived harm and addictiveness, but patterns differed by TNP.

Across the TNPs, nearly a quarter of the sample were categorized as current users and another quarter were classified as willing non-users, suggesting that prevention and cessation efforts are needed at this age. While TNP prevention interventions are usually implemented during early adolescence^26^, adolescents aged 14–15 years could benefit from efforts to prevent nearly a quarter of the sample, the willing non-users, from initiating TNP use.

Perceptions of harm and addictiveness were often highest for non-willing non-users, followed by willing non-users compared to users, consistent with previous studies that have found that perceptions of risks and benefits of smoking predict future initiation^27^. Interventions to correct cognitive misperceptions to prevent initiation among this high-risk group are needed given that willing non-users had significantly lower levels of perceived harm and addictiveness compared to non-willing non-users across a number of products.

Adolescents were less likely to report not knowing the harm and addictiveness of cigarettes and e-cigarettes compared to the other products. Given that most anti-TNP messaging for youth in the US is focused on cigarettes and e-cigarettes, the findings are not surprising^28^. However, they do raise an important gap in current tobacco control efforts, namely that adolescents may not be receiving harm and addictiveness messages for all currently available TNPs.

Our results highlight the importance of considering the range of currently available TNPs to develop relevant prevention and cessation interventions for adolescents. For example, perceptions of hookah’s addictiveness and harm were much lower compared to other TNPs. Previous studies have found similar findings, and these cognitive misperceptions were associated with smoking hookah^29^. Thus, interventions should address harm and addictiveness of all TNPs through targeted media campaigns to decrease the odds that these products will be used by adolescents who may not realize how harmful or addictive they are to their health.

Interestingly, peer use was associated with lower perceptions of addictiveness for pipe and cigarillos, while there were no observed effects of peer use on perceptions of harm. These findings should be explored in future studies to understand the role of normative influences in relation to future use. Additionally, male students reported lower odds of perceived harm for smokeless tobacco, pipe, cigarillos, and cigars, suggesting sex differences in the perceptions of TNPs, which might influence males to initiate these products. Black students reported lower perceived addictiveness for all TNPs, but there were no differences in perceived addictiveness. Overall, these demographic findings suggest that a precision health approach to TNP prevention and control is needed to address the varied perceptions and use patterns across all TNPs to prevent initiation and promote cessation.

For decades, schools have been a primary setting for delivering youth TNP prevention and control interventions, and there is now a large body of research demonstrating the effectiveness of school-based TNP prevention programs^26^. However, many of these interventions focus exclusively on one TNP, and those that address multiple TNPs do not target the range of currently available TNPs^26^. Additionally, most school-based programs address TNP prevention, but there is scant research indicating the long-term effectiveness of TNP cessation programs for youth^30^. In a recent Cochrane review, the authors could not identify a youth smoking cessation program that was superior to quitting unaided^30,31^. Similar to prevention programming, to our knowledge, most available cessation programs in the US only target a single TNP product (e.g. cigarettes or e-cigarettes)^32^, despite the variety of tobacco products on the market, and the evidence that many youth are using multiple tobacco products^2^. Our results suggest a need for both prevention and cessation interventions targeting the range of currently available TNPs, and tailored to the unique perceptions and willingness of adolescents to ensure that students receive messaging that will help them lead nicotine-free lives. Utilizing a mix of both teacher-led and asynchronous digital health instruction could overcome existing barriers to previous TNP interventions^33^. Specifically, a whole group teacher-led intervention could shift normative beliefs about the TNP use among peers, while the asynchronous digital health intervention could provide targeted instruction based on a student’s specific willingness to use different TNPs.

### Limitations

The current study was not without limitations. Since the findings were cross-sectional, we cannot say whether perceptions of harm and addictiveness will influence tobacco initiation among non-users in the current sample. Additionally, the sample was from two regions of Virginia, and thus results might not generalize to adolescents in other regions of the world. Finally, students were provided with a $5 gift certificate for participating in the study, which could have contributed to their willingness to participate.

## CONCLUSIONS

To our knowledge, this was the first study in the US to examine willingness and perceptions of harm and addictiveness of the range of TNPs currently available. Almost half of adolescents were either willing to use TNPs or were currently using them. Additionally, perceptions of willingness, harm and addictiveness varied by product and other demographic factors. These findings highlight the need for continued TNP prevention and cessation programs utilizing a precision health approach that addresses contemporary patterns of TNP susceptibility and use.

## Data Availability

The data supporting this research are available from the authors on reasonable request.
